# Human papillomavirus genotype distribution in tonsil cancers

**DOI:** 10.1186/1758-3284-3-6

**Published:** 2011-02-07

**Authors:** Jean Lacau St Guily, Christine Clavel, Claire Okaïs, Jean-Luc Prétet, Agnès Beby-Defaux, Gérard Agius, Philippe Birembaut, Anne-Carole Jacquard, Yann Léocmach, Benoît Soubeyrand, Didier Riethmuller, François Denis, Christiane Mougin

**Affiliations:** 1 Service d'Oto-rhino-laryngologie et chirurgie cervico-faciale, Hôpital Tenon, Université Paris 6 et Faculté de Médecine Pierre-et-Marie Curie, Assistance Publique-Hôpitaux de Paris, Paris, France; 2 INSERM UMRS 903, CHU Reims, Université de Reims, Laboratoire Pol-Bouin, Reims, France; 3 Sanofi Pasteur MSD, Lyon, France; 4 Univ Franche Comte, Besançon, France; CHU Besançon, Besançon, France; 5 CNRS UMR 6187, CHU de Poitiers, Laboratoire de Virologie, Université de Poitiers, Poitiers, France; 6 Service de Gynécologie-Obstétrique, CHU Saint Jacques, Besançon, France; 7 Laboratoire de Bactériologie et Virologie, CHU Dupuytren, Limoges, France

## Abstract

**Background:**

The incidence of tonsil cancers has increased in several countries. French data on HPV prevalence in tonsil cancers are scarce. The objective of this study was thus to assess the overall and type specific HPV prevalence in tonsil histological samples.

**Methods:**

This French retrospective multicenter study involved 12 centres located throughout the country. Were included 185 histological samples collected from year 2000 to 2009 with a validated diagnosis of tonsil invasive carcinomas. HPV prevalence was studied according to gender, age and histological type of cancer.

**Results:**

Overall HPV prevalence was 57% in tonsil cancers. Mean age of diagnosis was comparable in HPV positive tonsils cases (60 ± 11.2) and HPV negative tonsil cases (59 ± 9.6). HPV prevalence was significantly higher in female than in male cases (28/35 versus 78/150 in tonsil cases, respectively, P = 0.003). About 53% of tonsil cases were infected by a single HPV type. Only eight (4%) samples were infected by more than one HPV type. Among HPV positive samples, HPV 16 was found in 89% of tonsil cases. All other HPV types had prevalence below 5%.

**Conclusions:**

Our results indicate that HPV is common in tonsil carcinomas and emphasize the predominant role of HPV 16.

## Background

Head and neck cancers (HNC) are the fifth most common cancer worldwide with more than 600,000 cases diagnosed each year [1]. France is the country the most affected by HNC in Europe with more than 16,000 new cases in 2005 and 5,400 deaths. More cases are diagnosed in males than in females [2]. They are almost exclusively head and neck (HN) squamous cell carcinomas (SCC), observed in different anatomical sites: oral cavity, oropharynx, hypopharynx, and larynx [3]. Tonsil cancer incidence was estimated to be more than 1,700 in France in 2004 [4].

The overall incidence of HNC has fallen in recent years. This seems to be consistent with the decrease in tobacco and alcohol consumption. By contrast, the incidence of cancers in particular anatomical sites has increased [3]. The incidence of oropharyngeal tumours rose in the USA by 1.3% for base of tongue cancers and by 0.6% for tonsil cancers every year between 1973 and 2004 [5]. Tonsil squamous cell carcinomas (SCC) in the Stockholm area rose from 0.74/100,000 person-years in 1970-1979 to 1.65/100,000 person-years in 2000-2006 [6]. The age-adjusted incidence of tonsil cancer increased 3.5-fold in women and 2.6-fold in men between 1970 and 2002 in Sweden [7].

Well known main risk factors for developing oropharyngeal cancer are tobacco usage and alcohol consumption [3]. Socio-economic deprivation [8] and poor oral health [9] have also been reported. However a new profile of patients suffering from HNSCC is emerging. A better survival of patients with HPV positive HNSCC is observed [10]. An increase of oropharyngeal SCC in individuals with no history of tobacco or alcohol use has been described [3]. New risk factors such as sexual behaviors in HPV positive cancers are observed [11]. Different authors reported the presence of HPV in oropharyngeal tumors, especially in the lingual and palatine tonsils [3,6]. Augmented incidence of HPV-associated tonsil cancers seems to represent an emerging public health problem.

French data on HPV prevalence in tonsil cancers are scarce. The objective of this study was thus to assess the overall and type specific HPV prevalence in tonsil histological samples.

## Methods

### Study design

The overall study which involved oropharyngeal and oral cavity invasive cancers is described elsewhere. We present here results of a substudy focused on tonsil cancers. This French retrospective multicenter study involved 12 centres located throughout the country. Selection of the centres was mainly based on their willingness to participate trying to get most French geographical areas represented. To be included, histological samples had to be collected after year 2000. Each sample with a validated diagnosis of tonsil cancers and fixed in 4% buffered formalin before paraffin embedding were retrospectively included. Cases with diagnosis other than SCC were excluded. Each centre decided whether its own cases could be included. Patient data such as age at diagnosis, gender, area of residence, year of sample collection were collected for each case when available.

According to the French legislation (Public Health Code modified by the law n° 2004-806, August 9, 2004 and the Huriet-Sérusclat act 88-1138, December 20, 1988) and since this study only involved data extracted from medical records and stored histological specimens, no informed consent from the patients was necessary. Data collected from participating pathology centres were strictly anonymous.

### HPV genotyping

HPV genotyping was centrally performed using the INNO-LiPA HPV Genotyping extra test (Innogenetics Inc, Gent, Belgium). This kit allows the detection of 28 HPV types, 15 high-risk (HR) and 13 low-risk (LR) [12]. In order to be consistent with previous EDiTH studies [13,14] the 28 HPV genotypes identified by the INNO-LiPA assay were classified as follows: LR HPV: genotypes 6, 11, 26, 40, 43, 44, 53, 54, 66, 70, 71, 73 and 74; HR HPV: genotypes 16, 18, 31, 33, 35, 39, 45, 51, 52, 56, 58, 59, 68, 69 and 82

### Statistical analysis

The HPV genotype-specific prevalence was expressed as the proportion of HPV-positive samples among all carcinoma cases and among HPV positive cases. HPV prevalence was studied according to gender, age and histological type of cancer. Prevalence of HR and LR HPV was also calculated. Categorical variables were studied using 2-sided Chi-square or Fisher exact test when necessary. All statistical analyses were performed using SPSS v.11.5 for Windows. A p-value below 0.05 was considered as statistically significant.

## Results

A total of 220 of histological samples of tonsil invasive SCC were retrospectively collected from 12 French pathology centres scattered throughout the country. Patients were recruited throughout France: 28% in Paris area, 8% in the West area, 35% in the North and North East, and 28% in the South of France. 84% (185/220) were included in the study while 16% (35/220) were excluded from the analysis because neither HPV nor cellular DNA could have been amplified. Mean age at diagnosis was 60 ± 10.5 years. There were 81% (150/185) of males.

**Figure 1:**
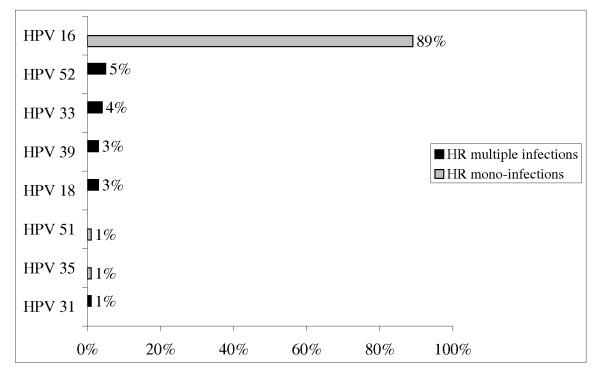
**HR HPV distribution among tonsil HPV positive cases (n = 106)**.

We found an overall HPV prevalence of 57% (106/185). Mean age of diagnosis was comparable in HPV positive tonsil cancer cases (60 ± 11.2) and HPV negative tonsil cancer cases (59 ± 9.6). HPV prevalence was higher in female than in male cases (80% (28/35) versus 52% (78/150) respectively, p = 0.003). About 53% (98/185) of tonsil cancer cases were infected by a single HPV type. Only 4% (8/185) of tonsil samples were infected by more than one HPV type, with at least one HPV HR. HPV 16 was found in 89% (94/106) of HPV positive tonsil cases. All other HPV types had prevalence below 5%. HPV 16 and/or 18 was found in 91% (96/106) of HPV-positive tonsil cases. (Figure 1) Year of sample varied from 2000 to 2009 (median of 2005) with no clear variation of the percentage of HPV-positive tonsil cancers with time.

## Discussion

HPV has been suggested to be associated with approximately 25% of HNSCC. HPV has been identified in the oropharynx and particularly in tonsil cancers in the literature [3,15]. Our study of 185 cases of tonsil SCC is among the largest European patient series presented with HPV analyses [16]. Tonsil cancers had a high prevalence positivity (57%) and a very low HPV-HPV multiple infection proportion (4%) compared with carcinomas from all other location within the head and neck region (data not shown) which agrees with previous studies [16,17]. Hannisdal et al identified the HPV in 52% (71/137) of patients with tonsil carcinomas from 1960 until 1996 in Norway [17]. Syrjanen et al found in their review that 51% of the 432 tonsil cancers were HPV-positive, and less than 5% were infected by multiple types of HPV [16]. Mellin et al showed from 1984 to 1996 that HPV was present in 43% (23/60) of tonsil cancers [18]. HPV was identified in 46% of patients with tonsil SCC (24/52) from 1987 to 1995 in the USA [19]. Inversely Li et al did not identify HPV in 16 tonsil cancer specimens from Chinese patients [20] whereas the positivity rate was 46% in Australian patients using the same methodology [21]. Chien et al found the HPV in 13% (14/111) of the patients with tonsil carcinomas in Taiwan from 1992 to 2005 [22]. The discrepancy in HPV prevalence could be partly explained by sample sizes, by the methods used to determine HPV prevalence (specimen type, method and duration of sample storage, HPV detection procedure), by the fact that HPV-positive cancers seem to increase during the last decades and by epidemiological and lifestyle differences, such as alcohol and tobacco consumption or sexual behavior.

Some authors found that the proportion of HPV positive tonsil SCC increased during the last decades. The proportion of HPV cancers in the county of Stockholm, using the same diagnostic method, was 23% (7/30) in the 1970s, 29% (12/42) in the 1980s, 57% (48/84) in the 1990s and 68% (32/47) in 2000-2002 [7] and 85% in 2003-2007 [6]. Hannisdal et al found that HPV positivity was significantly more frequent (64%) in the latter period (1985-1996) of the patient series compared with 38% in the first period (1960-1985) [17]. We did not observe any variation during the study but our study period was too short to demonstrate any evolution with time.

HPV 16 was the genotype the most identified in the literature, 93% (77/83) of HPV-positive cases in the series of Nasman et al 2003-2007 [6]. As for the review of Syrjanen, HPV 16 had been identified in 84% of the 216 HPV DNA positive tonsillar SCCs [16]. Consistently with these data, we found in our study that HPV 16 was present in 89% of all HPV-positive tonsil cancers. Interestingly, a meta-analysis stratified by anatomical site suggested that the association between HPV 16 and cancer was strongest for tonsil (OR 15.1 [6.8-33.7), intermediate for oropharynx (OR 4.3 [2.1-8.9]) and weakest for oral (OR 2.0 [1.2-3.4]) and larynx (OR 2.0 [1.0-4.2]) [23].

In our series, HPV prevalence was higher in female than in male cases (80% versus 52%) even if men accounted for the majority (81%) of the total tonsil cancers, which was concordant with some studies. Nasman et al found that HPV was present in 95% (21/22) of women cases of tonsil cancers and in 82% (62/76) of men cases of tonsil cancers [6]. HPV was identified in 65% of women cases (11/17) and in 35% (15/43) of men cases in Sweden[18]. Chien et al also noted that the greatest incidence of HPV-positivity was associated with females (p < 0.001, odds ratio = 18.48, 95% CI = 5.55-61.51) [22].. Risk factors for HPV positive and negative HNC such as sexual behavior for HPV positive tumors and tobacco and alcohol for HPV negative tumors were clearly identified in various studies [3,11]. Thus, the higher proportion of HPV observed in women cases could possibly result from a different risk factor distribution between men and women since smoking and alcohol consumption are known to be more frequent in men [24]. However, these points cannot be addressed in our study since data were extracted from pathology records and we had no patient information concerning alcohol and tobacco habits and sexual behaviour.

Different authors found that HPV positive carcinoma was associated with younger patients. Nasman et al observed that patients with HPV positive tonsillar cancer were younger than patients with HPV negative cases (59 years versus 68 years) [6]. This trend was not observed in our study.

Our study has limitations. First since the recruitment was rather heterogeneous between centres and centres were not evenly scattered on French territory despite patients were recruited throughout France, the study sample can not be considered as fully representative of the French tonsil cancer population. Secondly, we used DNA detection only; in this retrospective study it was not possible to determine the presence of transcripts. However, Nasman et al found that HPV 16 E6 or E7 RNA were expressed in the majority of the analysed HPV 16 positive tumors [6]. Syrjanen et al found in their review that HPV-16 E6 and E7 are actively transcribed in most of the analysed tonsil carcinomas [16]. Hence, our results seems to be realistic even if we only assessed DNA detection.

## Conclusions

Our results indicate that HPV has a high prevalence in tonsillar SCC. We emphasize the predominant role of HPV 16 since 89% HPV-positive tumors were linked with HPV 16; moreover 91% were associated with HPV 16 and/or 18. Unlike for cervical cancer, the association of HPV and oncogenic mechanisms remain to be clarified. These results are an indicator of the HPV distribution in tonsil cancers before HPV vaccine implementation among women. The HPV vaccination of young women will be able to contribute *a posteriori *to clarify the possible causal association between tonsil cancers and HPV.

## Competing interests

- Jean Lacau Saint Guily, MD: Member of the scientific EDiTH VI advisory board

- Claire Okaïs, PharmD, is an employee of Sanofi Pasteur MSD

- Anne-Carole Jacquard, PhD is an employee of Sanofi Pasteur MSD

- Jean-Luc Prétet, PhD: occasional speaker for SPMSD

- Agnès Beby-Defaux, MD, PhD: The author declares that he has no competing interest

- Christine Clavel, PharMD, PhD, belongs to the steering committee of MERCK about vaccines

- Gérard Agius: The author declares that he has no competing interest

- Philippe Birembaut, MD: The author declares that he has no competing interest

- Yann Leocmach, MD, is an employee of Sanofi Pasteur MSD

- Benoît Soubeyrand, MD, is an employee of Sanofi Pasteur MSD

- Didier Riethmuller, MD, PhD: expert for SPMSD

- Christiane Mougin, MD: Travel support

- François Denis, MD, PhD: The author declares that he has no competing interest

## Authors' contributions

JLSG participated to the study design, data analysis and interpretation and manuscript preparation and review. CC participated to the study design, data analysis and interpretation and manuscript preparation and review. CO participated to the data analysis and interpretation and manuscript preparation and review. JLP participated to the study design, data analysis and interpretation and manuscript preparation and review. ABD participated to the study design, data analysis and interpretation and manuscript preparation and review. GA participated to the study design, data analysis and interpretation and manuscript preparation and review. PB participated to the study design, data analysis and interpretation and manuscript preparation and review. ACJ participated to the study design, data analysis and interpretation. YL participated to the study design, data analysis and interpretation and manuscript preparation and review. BS participated to the study design, data analysis and interpretation and manuscript preparation and review. DR participated to the study design, data analysis and interpretation and manuscript preparation and review. FD participated to the study design, data analysis and interpretation and manuscript preparation and review. CM participated to the study design, data analysis and interpretation and manuscript preparation and review. All the authors have given approval of the version to be published.
